# Efficacy of an online lung ultrasound module on skill acquisition by clinician: a new paradigm

**DOI:** 10.3389/fped.2024.1406630

**Published:** 2024-06-11

**Authors:** Alok Sharma, Gunjana Kumar, Rema Nagpal, Kirti Naranje, Arnab Sengupta, Vanitha Jagannath, Sonali Suryawanshi, Pradeep Suryawanshi

**Affiliations:** ^1^ Department of Neonatal Medicine, Corniche Hospital, Abu Dhabi, United Arab Emirates; ^2^ Department of Neonatology, National Institute of Medical Sciences and Research, Jaipur, India; ^3^ Department of Neonatology, Bharati Vidyapeeth University Medical College, Hospital, and Research Centre, Pune, India; ^4^ Department of Neonatology, Sanjay Gandhi Postgraduate Institute of Medical Sciences, Lucknow, India; ^5^ Department of Pediatrics, University of Toledo College of Medicine, Toledo, OH, United States; ^6^ Department of Pediatrics, American Mission Hospital, Manama, Bahrain; ^7^ Department of Pharmacology, Bharati Vidyapeeth University Medical College, Hospital, and Research Centre, Pune, India

**Keywords:** lung ultrasound, point-of-care ultrasound, survey, online curriculum, respiratory pathology

## Abstract

**Introduction:**

Lung ultrasound (LUS) as an assessment tool has seen significant expansion in adult, paediatric, and neonatal populations due to advancements in point-of-care ultrasound over the past two decades. However, with fewer experts and learning platforms available in low- and middle-income countries and the lack of a standardised supervised training programme, LUS is not currently effectively used to the best of its potential in neonatal units.

**Methodology:**

A cross-sectional survey assessed the efficacy of learning LUS via a mentor-based online teaching module (NEOPOCUS). The questionnaire comprised the clinicians’ demographic profile, pre-course skills, and self-assessment of skill acquisition after course completion with ongoing hands-on practice.

**Results:**

A total of 175 clinicians responded to the survey, with the majority (87.9%) working in level 3 and 4 neonatal intensive care units. Clinicians had variable clinical experience. Of them, 53.2% were consultant paediatricians/neonatologists with over 10 years of experience. After the course, there was a significant increase in clinician confidence levels in diagnosing and assessing all LUS pathology, as evidenced by the increase in median cumulative scores [from baseline 6 (interquartile range, IQR, 6–9) to 20 (IQR 16–24), *p* < 0.001] with half of them gaining confidence within 3 months of the course.

**Conclusion:**

An online curriculum-based neonatal lung ultrasound training programme with clinician image demonstration and peer review of images for image optimisation increases self-reported confidence in diagnosing and managing neonatal lung pathology. Web-based online training in neonatal lung ultrasound has merits that can help with the delivery of training globally, and especially in low- and middle-income countries.

## Introduction

Point-of-care ultrasound (POCUS) is an emerging imaging modality in critical care medicine. Integrating POCUS with the clinical presentation of a sick patient represents a transformative change in conventional practice, especially in the intensive care unit. Being a real-time bedside tool with no risk of radiation exposure, it is intended as an adjunct alongside clinical correlation in the management of small and sick neonates. It is used both as a diagnostic aid and to support therapeutic procedures. It enables bedside clinicians to make decisions in real time (1, 2).

The use of POCUS by neonatal intensive care clinicians has been dramatically augmented in recent years, with the acquisition of skills through certified training programmes (1–4). In neonates, lung ultrasound (LUS) is emerging as a relatively more recent skill that has paved the way for early and timely diagnosis of various respiratory disease states, such as pneumothorax, pleural effusion, pneumonia, and respiratory distress syndrome (RDS). Functional applications include LUS scoring for the management of RDS. Using LUS can reduce the time needed for management decisions, such as the need for surfactant in neonates with RDS. It can also aid intercostal drainage in the case of babies with pneumothorax and pleural effusion. LUS can be beneficial in units that lack the facility of bedside X-rays. It can help prevent the transportation of the sickest and smallest neonates to radiology in such circumstances. Sonography of the lung can be performed quickly, minimising handling of the baby and avoiding radiation (2).

Being primarily an operator-dependent tool, learners must have effective training with a robust learning curve. This must be facilitated via well-designed LUS training programmes covering an established comprehensive curriculum. The curriculum content should be designed to maximise learning and skill acquisition while ensuring patient safety (5–7). The intention should be to impart relevant knowledge and skills to the learner that enable clinical practice with a defined framework, standards, and governance. The training module should be based on adult learning principles (Kolb's cycle) and be learner-friendly and achievable. The limitations of performing LUS should be known to learner physicians. The intention is not to take over the radiologist's role but to assist in real-time bedside assessment of the disease process, evaluate the response to treatment, and assist procedures in the absence of these full-time specialists (8, 9).

Such standards have now been developed by learned societies and international bodies in neonatology, such as the American Academy of Paediatrics (AAP) (10), the European Society of Paediatric and Neonatal Intensive Care (ESPNIC) (11), and others (12). The real question, then, is how best to deliver lung ultrasound training in practice to allow learners to perform LUS safely. The learning curve has yet to be established in neonatology.

Existing training programmes in LUS include those delivered face-to-face and via e-learning (10–13). Some challenges of the existing LUS training in neonatology are that courses are of short duration (1–2 days), cannot provide sufficient time for supervised hands-on training, and do not provide ongoing support after course completion. This does not give the participants the benefit of expert supervision and ongoing assessment of competency and/or experiential learning that is needed to master all the technical and diagnostic aspects of LUS as espoused in the standards above (10–12). Furthermore, not everyone can travel to attend face-to-face courses and/or train in dedicated neonatal LUS fellowships. These problems are magnified in low- and middle-income countries where there is not only a shortage of trainers locally, but the cost involved in getting such training abroad is prohibitively expensive. Online portals and training modules can allow learners worldwide to train in lung ultrasound remotely. They can connect, interact, share experiences with experts without travelling, and interact with other learners (14–16). The question remains whether such training allows participants to gain experiential learning in LUS, which is needed to perform, interpret, and make decisions in sick neonates confidently.

The aim of the present study was to investigate the impact of online lung ultrasound training with mentoring on the acquisition of skills and to assess learner confidence in applying these skills and making decisions based on them after completing such training.

## Methodology

LUS training was imparted to learners as part of an online POCUS module (NEOPOCUS) conducted by an expert in the field of POCUS based in India over 6 months. The expert who delivered the module is a neonatologist with 24 years of experience in neonatology, a member of the Neonatologist Performed Echocardiography (NPE) group of the European Society of Paediatric Research, with more than 15 years of experience in POCUS. He has been the chairperson of the Ultrasound and Echocardiographic Committee of the National Neonatology Forum (NNF) since 2012.

The other modules in the POCUS module (NEOPOCUS) included cranial US, gut US, renal US, echocardiography, assessment of lines, and use of US in procedural skills such as lumbar puncture. The LUS module was conducted over 1 month (eight sessions). It comprised a curriculum consisting of didactic teaching (Terminology, Ultrasound Physics, Knobology, Machine Settings), recognition of the normal lung (terminologies such as lung sliding, A and B lines and all relevant signs), and recognition of lung pathology [RDS, transient tachypnoea of newborns (TTNB), pneumothorax, pleural effusion, consolidation, atelectasis, and pneumonia]. Additional sessions covered LUS scoring and procedures like chest drain insertion. The course was designed as two sessions per week, each with a duration of 120–150 min. The total duration was 16 h. The initial hour of each session, after the introductory section, was spent on peer review of the images presented by the clinicians of lung ultrasound scans performed in their respective units. The intention was to help participants consolidate their theoretical learning and practical experience in these live face-to-face sessions. This was aided by expert critique to improve image acquisition, quality, and interpretation. The latter half of the session covered the next part of the curriculum. Participants also learnt from other colleagues’ images, enabling a review of various neonatal lung conditions with clinical correlation. This helped with experiential learning and fulfilled elements of Kolb's cycle of adult learning.

Four batches have been trained over 2 years (January 2021 to December 2022), comprising 185 clinicians. The four courses were similar in content, delivery of education, and time for practice and peer review. The lead expert specialist delivered peer review across all the batches. In early 2024, we conducted a cross-sectional survey over 2 weeks. We invited all the participants from four batches for their feedback regarding their learning, skill acquisition from the course, confidence in diagnosing pathology, and the utility of lung ultrasound in decision-making. All the course participants were practising neonatologists/paediatricians interested in neonatology, with the majority having access to point-of-care ultrasound at their respective centres.

The questionnaire included demographic data, information regarding their pre-course skills, self-assessment of confidence in performing LUS, and ability to diagnose, report, and make management decisions for each disease state in the neonatal intensive care unit (NICU) (Supplementary File S1). The survey was initially evaluated for ease of completion and feasibility by seven practising neonatologists. After further modifications, the final version was disseminated to all the clinicians of the four batches by email using an online survey tool, with a brief background and purpose for conducting this study. Clinicians were invited initially, with two subsequent reminders. Missing data were excluded from the final analysis for that specific question. Consent was implied for participation in the survey. All data received were kept confidential. No incentives were offered to participate in the survey.

## Statistical analysis

For analysis of responses and scoring the confidence level for each lung pathology, we utilised a Likert scale in the range of 1–5, with 1 indicating no confidence and 5 representing the highest confidence level. The scoring was also applied to other responses and a biostatistician completed an analysis.

The normality of the continuous variables was assessed using the Kolmogorov–Smirnov test, and non-normally distributed continuous variables are presented as the median [interquartile range (IQR)]. Categorical variables are presented in frequency (%). The Wilcoxon signed-rank test was used to compare the median scores between baseline and post-observations. The association of the change groups with independent variables was evaluated using the appropriate chi-square test or Fisher’s exact test. All the results are presented at a minimum two-sided 95% confidence interval. Graphical representation of the variable was carried out using the bar diagram and box plot. All the statistical analyses were performed using the Statistical Package for Social Sciences version 23 (SPSS-23, IBM, Chicago, IL, USA).

## Results

**Figure 1:**
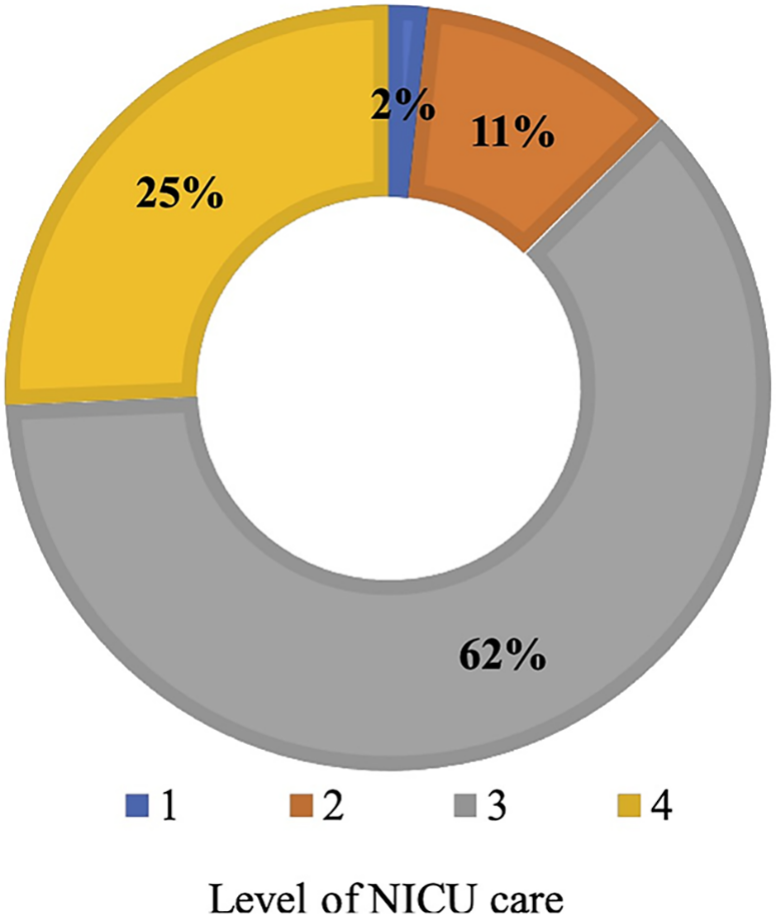
Levels of care in NICUs provided by clinicians.

**Figure 2:**
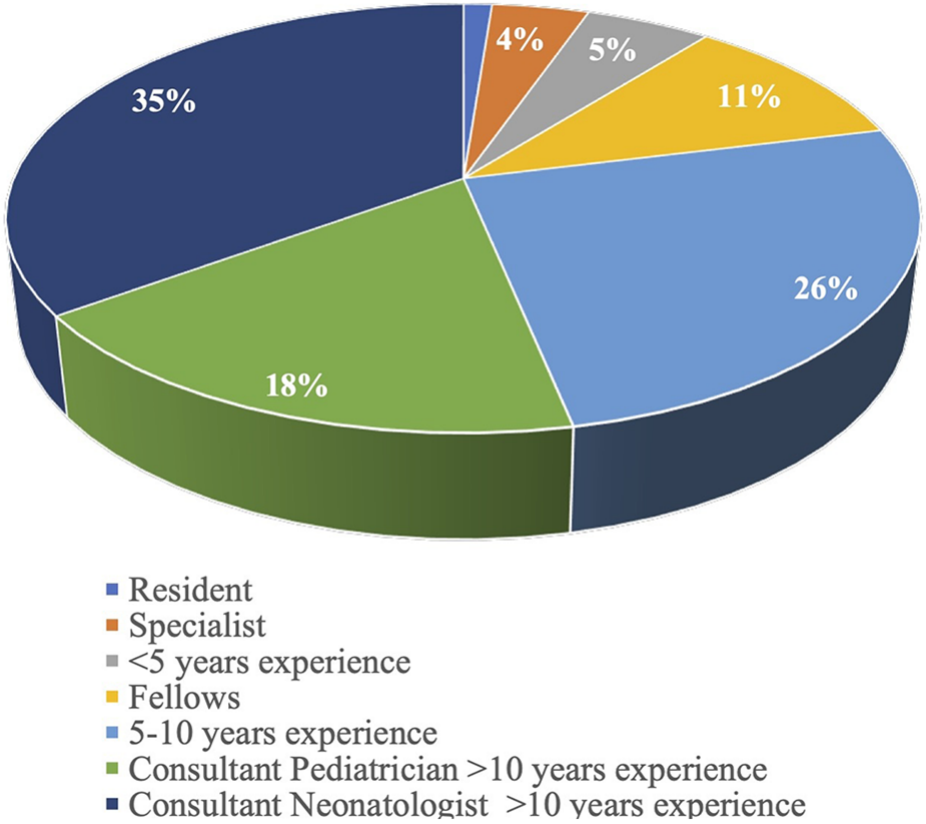
Clinical experience of survey clinicians.

In total, 175 (94.6%) respondents participated in the survey, of which all but one were practising doctors or medical specialists in neonatology or paediatrics with a neonatal interest. Of the respondents, 148 (84.6%) reported having complete access to point-of-care ultrasound services in their unit during the course. Most clinicians (87.9%) worked in level 3 and 4 NICUs, with only 2% and 11% stationed at levels 1 and 2, respectively (Figure 1). The clinical experience of the respondents varied, with 53.2% working as consultant paediatricians/neonatologists with more than 10 years of experience, 25.7% working as consultant senior specialists with 5–10 years of experience, and 21.1% working as trainees/registrars (Figure 2).

Only 4% of the survey participants reported that their institution had an adopted training pathway for accreditation in neonatal POCUS (cranial, lung, cardiac, or vascular access), whereas 26.9% had informal non-certified training (based on the principle of see, learn, do) with no specific curriculum. Most clinicians (69.1%) had no prior training in POCUS or LUS.

Despite the widespread use of POCUS across specialties globally, 65.9% of responders specified that adult or paediatric radiologists primarily performed and interpreted reported LUS in their respective units. Only 13.2% of such assessments were performed by trained neonatologists with or without accreditation. A further 21% did not perform LUS at all.

**Figure 3:**
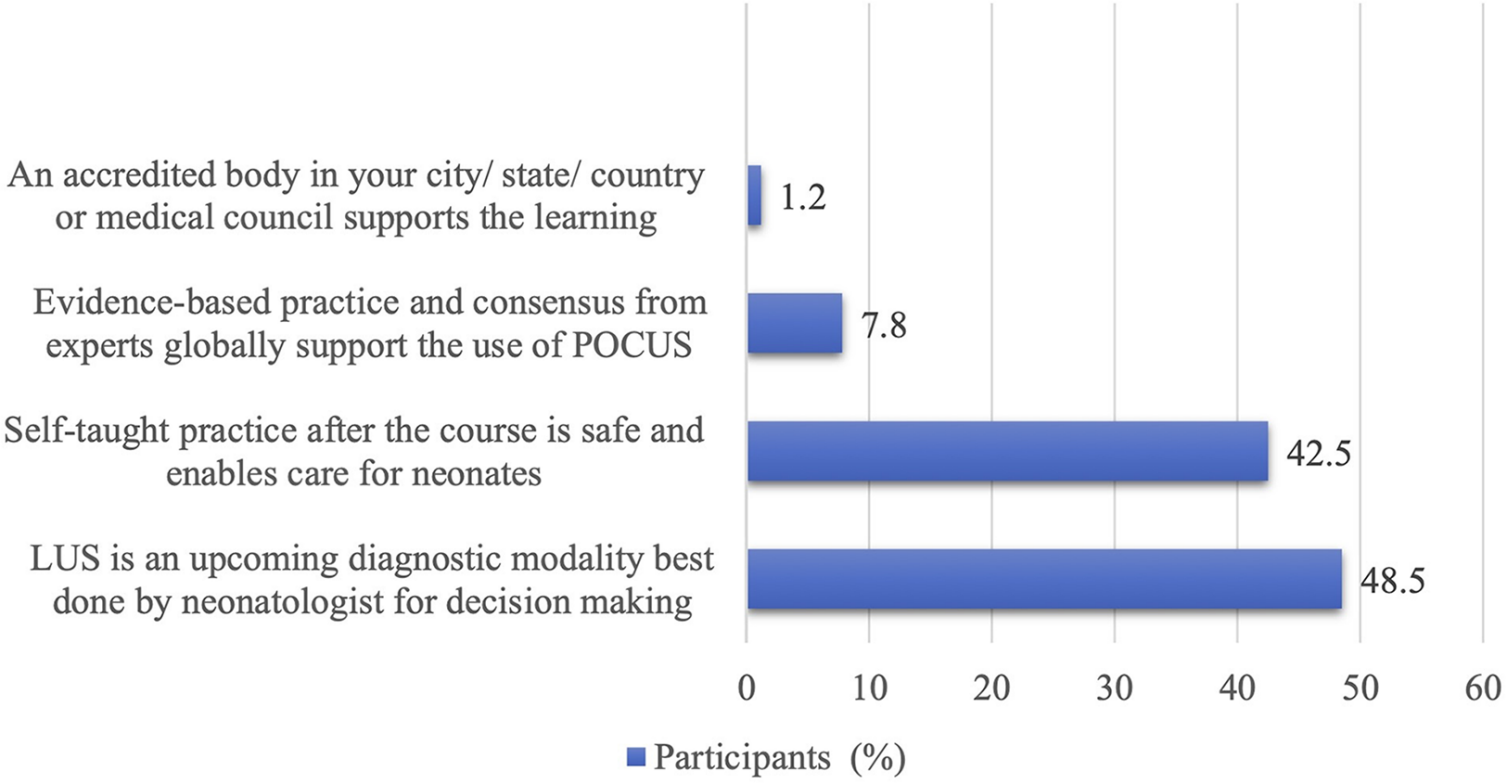
Clinician motivation for undertaking an online lung ultrasound course.

On explicitly being asked about their primary motivation for learning lung ultrasound, 48.5% of the clinicians perceived LUS as an upcoming diagnostic modality, allowing them to make diagnoses and bedside decisions. Of them, 42.5% expressed interest as they felt it would be safe and helpful in caring for sick and small neonates after the course, mainly where radiologists were unavailable. Of the respondents, 8% cited the global influence and expert consensus supporting POCUS as their driving factor. Only a small fraction (1.2%) felt that accreditation from local bodies was influential in pursuing such training (Figure 3).

## Pre- and post-course clinical experience with LUS

**Figure 4:**
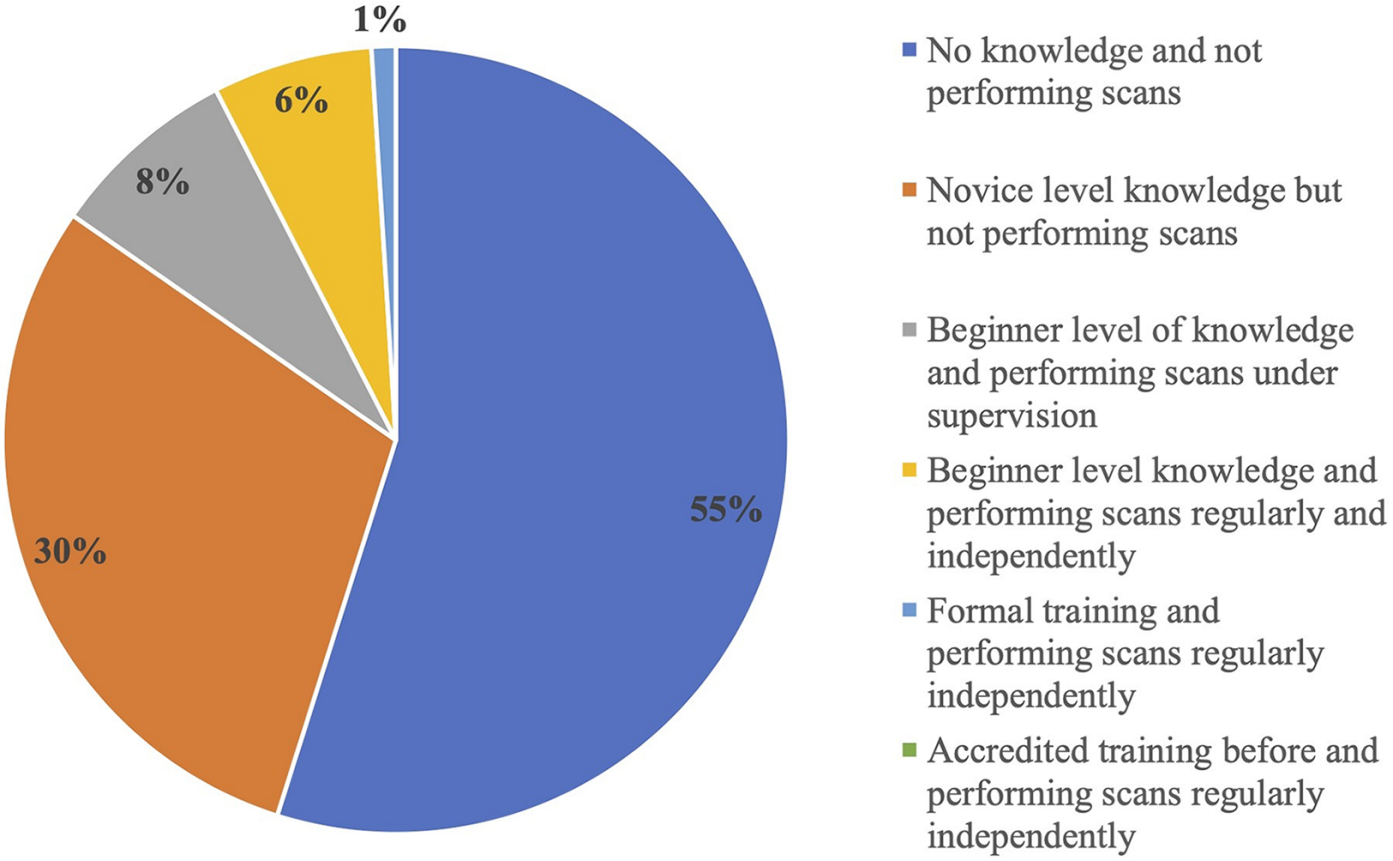
Pre-course clinician knowledge in lung ultrasound.

**Figure 5:**
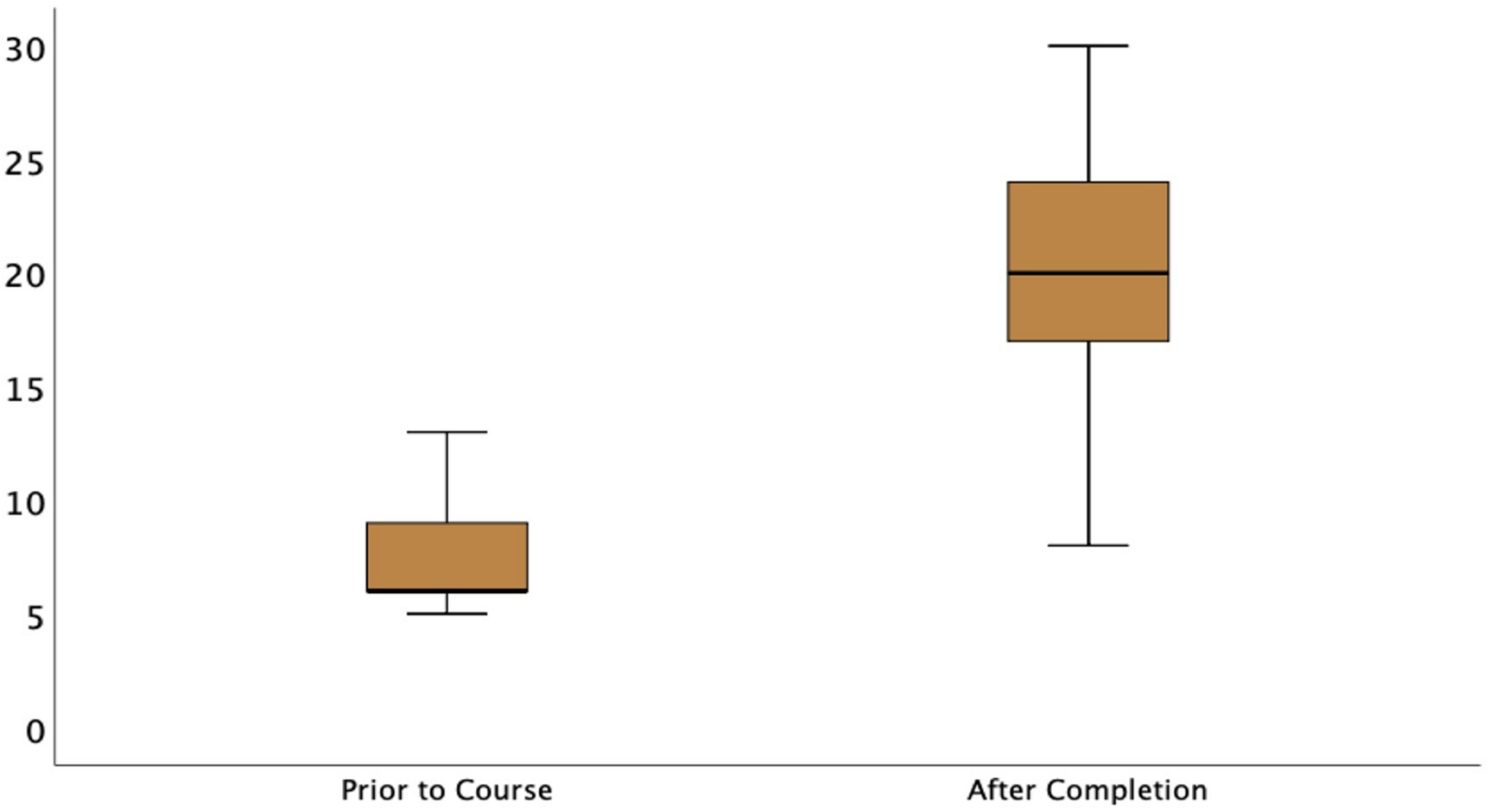
Boxplot showing cumulative pre- and post-course confidence scores of participants in diagnosis and assessment of lung pathology.

The confidence level of individual clinicians in diagnosing and assessing each LUS pathology was scored by themselves before and after the course. More than half of neonatologists (55%) were either not aware of the utility of LUS before the commencement of this course or needed more knowledge and were not performing the lung scans. Some of them (30%) had novice-level knowledge but were not performing scans, another 8% had beginner-level knowledge and were performing scans only under supervision, and a minority (6%) were performing scans regularly and independently despite having beginner-level knowledge with no formal training. Only 1% of the clinicians enrolled had undergone formal training courses (Figure 4). There was a significant increase in the confidence level of clinicians in the diagnosis and assessment of all LUS pathologies after the course, as depicted in the rise in cumulative score after the course [from baseline 6 (IQR 6–9)–20 (IQR 16–24), *p* < 0.001] (Figure 5 and Table 1).

**Table 1:** Total score of each participant for confidence in diagnosis and assessment of the all-LUS pathology before and after the course.

Variables	Pre-NEOPOCUS Course score	Post-NEOPOCUS score	*p*-value
Total score, median (IQR)	6 (6–9)	20 (16–24)	<0.001

**Figure 6:**
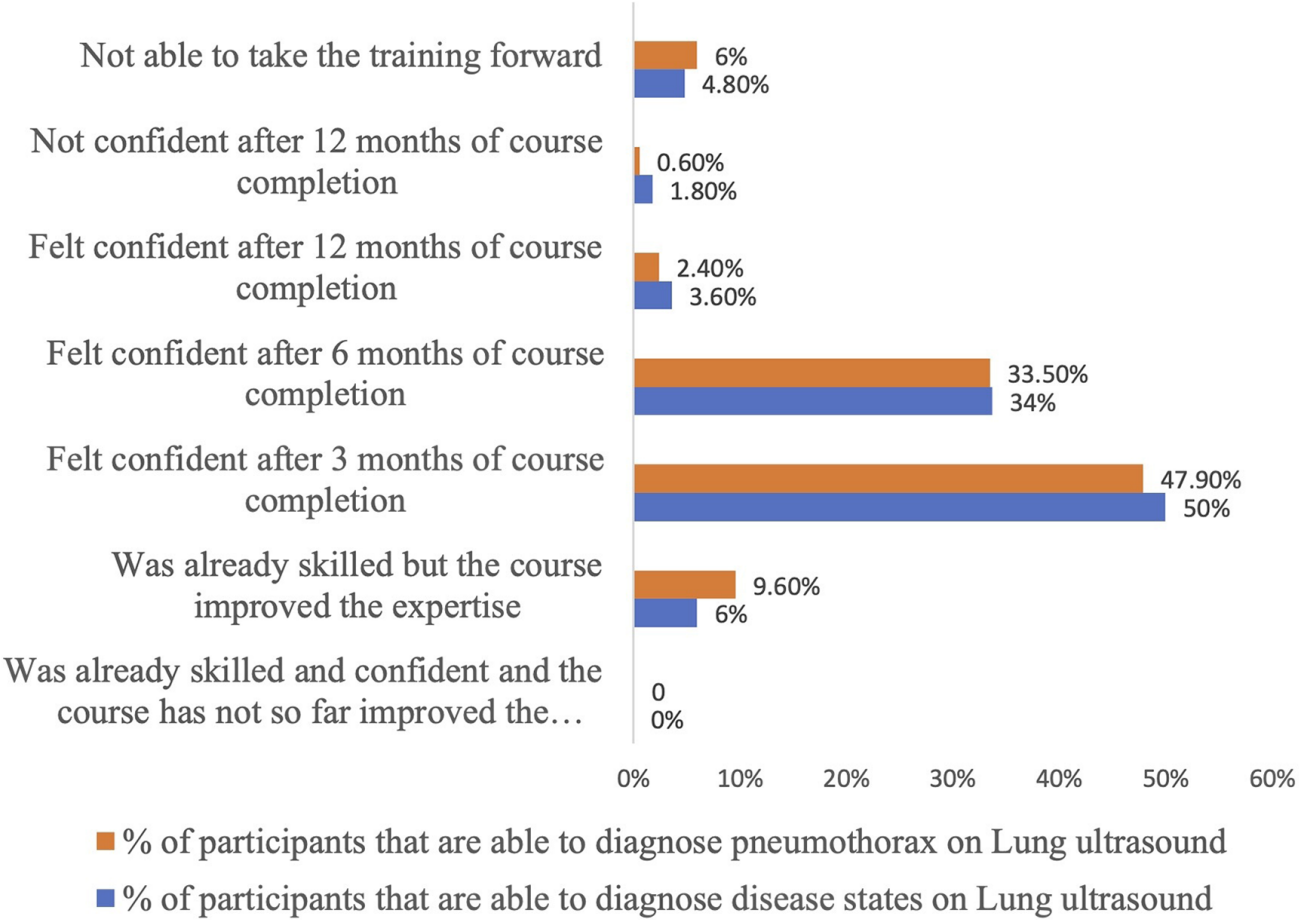
Post-course duration before independent reporting of lung ultrasound vs. pneumothorax.

A subgroup analysis was performed to examine the impact of clinicians’ prior experience levels on learning outcomes from the course. Interestingly, no significant differences were observed in learning outcomes across different experience levels, indicating that the course was equally effective regardless of prior experience (Supplementary Table S1). After 3 months of course completion, half of the participants were confident in diagnosing pneumothorax or other diseased states with LUS. However, just one participant reported that they did not gain confidence within a year, and some needed help to carry the training forward (6%). Approximately 34% of clinicians gained confidence 6 months after course completion, 3% required 12 months to acquire skills, and the rest, approximately 10%, claimed they were already skilled, but the course improvised the expertise (Figure 6).

The physician's confidence in diagnosing individual lung pathologies and making treatment decisions based on LUS has been depicted in Table 2. Significant differences were noted regarding most clinicians’ confidence in diagnosing individual lung pathologies before and after the course. This was shown by the decline in the number of clinicians who were not confident before versus those after the course in various diseased states, such as RDS (75.9% vs. 1.2%), TTNBs and pneumothorax (70.5% vs. 0.6%), and collapse/atelectasis (78.2% vs. 4.4%), with diaphragm assessment (83.1% vs. 9.8%) being more challenging.

**Table 2:** Pre- and post-course (self-evaluated) confidence level in diagnosis of disease.

Diseased state	Pneumothorax		RDS/TTNB		Consolidation/pneumonia		Collapse/atelectasis		LUS scoring for RDS		Diaphragm assessment	
Level of confidence												
	Pre-course (%)	Post-course (%)	Pre-course (%)	Post-course (%)	Pre-course (%)	Post-course (%)	Pre-course (%)	Post-course (%)	Pre-course (%)	Post-course (%)	Pre-course (%)	Post-course (%)
Not confident in diagnosis	70.5	0.6	75.9	1.2	73.6	1.2	78.2	4.4	78.8	1.2	83.1	9.8
Some confidence in diagnosis, need expert supervision	15.7	10.9	12.4	13.4	12.3	12.4	11.5	17.5	10.3	17.1	10.2	22.1
Confident in diagnosis, occasional expert supervision	9	32.1	8.6	29.9	9.8	29	8.5	35	7.9	26	5.4	27.6
Independent diagnosis, no supervision	2.4	39.4	1.2	37.8	3.1	40.1	0.6	31.9	1.8	39	0.6	30.1
Independent diagnosis and supervises others	2.4	16.7	1.9	17.7	1.2	17.3	1.2	11.3	1.2	16.5	0.6	10.4

**Figure 7:**
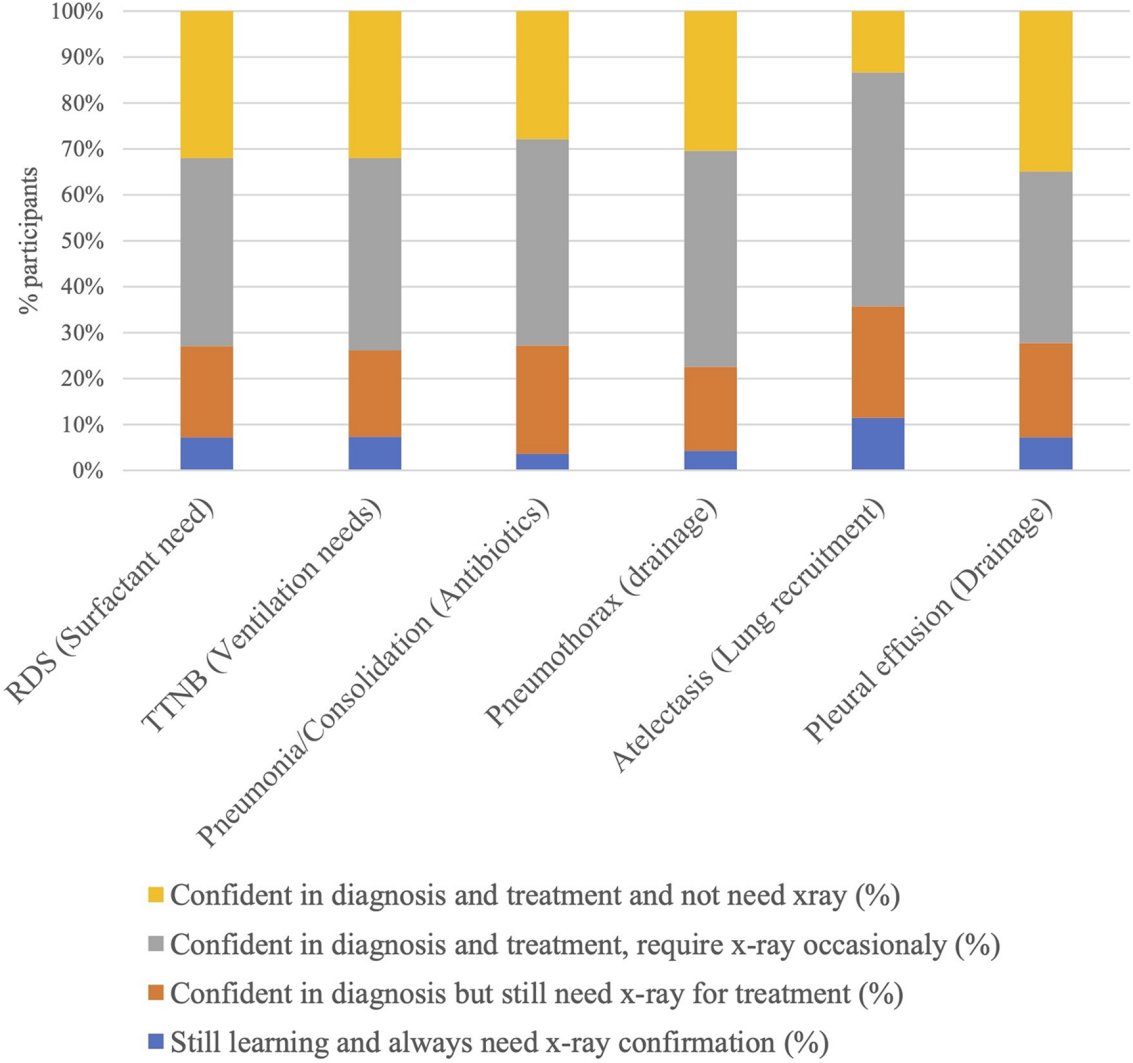
Post-course confidence in decision-making when using lung ultrasound and need for chest X-ray.

More importantly, participants found LUS helpful in making treatment decisions for different conditions. In RDS, for example, 72.9% of clinicians found neonatal LUS useful in the diagnosis and could make treatment decisions either without X-rays or occasional chest X-rays. Similarly, 73.9% of the clinicians found neonatal LUS useful in diagnosing pneumothorax and could treat the disease with or without the occasional need for chest X-rays. This indicated the added benefit of avoiding radiological exposure. Similarly, 72.7% of participants could diagnose and treat pneumonia/consolidation without or with the occasional need for an X-ray, and 72.3% of pleural effusions were managed without occasional X-rays (Figure 7).

## Discussion

Over the past decade, there has been a rise in the adoption of POCUS performed by neonatologists (17, 18). POCUS performed by a neonatologist/paediatrician differs from a comprehensive diagnostic evaluation by a radiologist, as the physician can perform and interpret the study, promptly integrate the information in the clinical context, and subsequently re-evaluate the clinical status. In addition, this can be done at the bedside and serial POCUS can be performed (11). In the current cross-sectional survey, most clinicians agreed that this was the primary factor influencing their desire to train in LUS.

Research shows that with its descriptive and functional applications, neonatologists trained in LUS can perform comprehensive scanning at the bedside and interpret the different pathologies accurately. In addition to the advantages mentioned above, research indicates that LUS has excellent sensitivity and specificity for diagnosing RDS, TTNB, pneumothorax, and meconium aspiration syndrome. Serial lung ultrasound may help evaluate surfactant needs in RDS, approximately assess the size of pneumothorax and pleural effusions, and help with intercostal drainage (10, 11, 19–21).

What still needs to be discovered and adequately researched is how best to provide neonatologists with adequate training to perform LUS safely while maintaining high standards in diagnosing and managing the different neonatal lung pathologies alongside clinical correlation. It is proposed that training programmes offer introductory didactic sessions through in-person lectures, podcasts, and website teaching modules. Learners must also acquire dexterity and competency while performing hands-on scans to implement bedside ultrasound effectively. This didactic and practical training overlap will assist learners in implementing LUS in practice, under supervision, and then independently (10).

Unfortunately, significant disparities persist in how training is currently delivered. This is not limited to training alone but also impacts clinical practice regarding indications, protocols, credentialing, and clinical governance. There is concern that this could affect its uptake globally (12, 22–24).

Previous surveys have not evaluated the impact of LUS systematic training programmes on practices in neonatal units. These surveys are studies of clinicians performing lung ultrasounds in high-income countries focusing on unit-based practice (22, 23). A recent survey of the practice of 560 neonatal units using LUS in Europe revealed variation in training being received by colleagues performing the procedure. In 223 (40%) units, clinicians attended a neonatal LUS course; in 62 (11%) units, clinicians attended a LUS course for paediatricians; and in 53 (9%) units, they participated in a general POCUS course. Clinicians in 119 (21%) units performing LUS received no specific training (24). There are no studies looking at the learning curve of learners in neonatal lung ultrasound from low- and middle-income countries.

We performed a physician-based survey to ascertain the individual's practice preferences and to evaluate the ramifications of an online, supervised learning programme of LUS in neonatal practice (Supplementary Material).

Our study population comprised formally trained neonatologists/paediatricians with a special interest in neonatology who worked in units that predominantly provided level 3 and 4 care (Figure 1). Data from the survey represents the practices of physicians working in units that offer an advanced level of care, where most (87%) have access to ultrasound machines whenever required. They tended to be established neonatologists/paediatricians with a particular interest in neonatology as opposed to trainees and residents (78.9% vs. 21.1%), with 69.1% having no formal training before this course. A recent European survey reveals a similar distribution with LUS performed by neonatologists as opposed to trainees and residents (24). We must understand the motivations for learners wanting to learn LUS in neonatology, as this may affect how training is provided to individuals keen to learn the skill. In this study, the prime motivator was the importance of LUS as an upcoming diagnostic modality, allowing neonatologists to use POCUS alongside clinical correlation to aid decision-making. The second motivator was the need for more skilled radiologists to perform and interpret LUS. In countries like India, paediatric radiologists may not be available to perform bedside LUS. Adult radiologists might not have trained in neonatal lung ultrasound.

Interestingly, its endorsement by learned societies and international bodies (10–12) and its use as additional accredited skills were ranked lower. This indicates clinicians want to use it primarily as a diagnostic tool in neonates rather than just a tick-the-box skill. We did not explore the barriers to uptake of LUS and its implementation in candidates who might have yet to take their training forward, which is a limitation of our study. The most significant barriers to implementing LUS identified by studies in developed countries are the lack of experience with the technical aspects of using the ultrasound machine, lack of experience with image interpretation, lack of qualified faculty for training, time constraints, and physician resistance to new technology (24, 25). Training in LUS with adequate supervision may help overcome these issues. It also helps maintain governance and patient safety while learners are getting skilled in performing, interpreting, and reporting LUS. While hands-on practical training has been recommended in the New International Guidelines and Consensus on the Use of lung Ultrasound, it is also acknowledged that remote mentoring of learners is possible (26).

This online 6-month platform addresses both the didactic and practical elements of LUS as per international recommendations (10, 12, 26). Learners are mentored online under expert supervision through a review of their images in live sessions. Enrolled physicians get an opportunity to demonstrate their images regularly during these sessions. There is a discussion of the cases and diagnosis, as well as suggestions on probe positioning and image optimisation. Candidates are also exposed to scans done by each other with different machines and probes that allow them to understand the importance of comprehensive imaging with the right equipment and the limitations of using what might be available in their setup. The mentorship sessions involve a discussion on diverse case scenarios, where an added advantage is that participants learn ultrasound protocols and standardised terminology for the different lung pathologies to ensure uniform practice. An essential aspect of POCUS is that proficiency in skills can diminish rapidly unless regular scanning is pursued, particularly among novice sonographers (27–29). Live recordings of the sessions are provided to the participants to review later, as they may need to refer to them while continuing to consolidate their skills. International guidelines do not currently endorse a minimum number of scans to maintain such skills (10–12, 26). We acknowledge that the number of learners had increased in the last cohort of learners, necessitating additional peer review sessions often facilitated with the help of learners from previous cohorts. A minimum number of lung ultrasound scans to be performed by each learner was not formalised in these cohorts.

In this study, most clinicians either had no prior experience or possessed only novice-level knowledge in this area. This observation underscores the need for a comprehensive approach to LUS training with didactic and practical training covering a structured curriculum. International recommendations specify a need for but have not formalised a neonatal-specific curriculum for LUS training (10–12, 24, 26). The curriculum used in this training module is summarised in the Supplementary Material.

Given that many clinicians were starting from a basic or inexperienced level, the online module was designed to cover fundamental principles of ultrasound physics, terminology, scanning techniques, protocols, diagnostic criteria for pathology, reporting, and clinical application. The peer review sessions, interactive learning modules, and case-based discussions enhanced proficiency and confidence in interpreting LUS examinations. Another limitation of this module was that a formal assessment was not part of this training module. Evaluation of theoretical knowledge and practical skills in lung ultrasound is well described in adult medicine (30). Interestingly, position statements by the AAP (10), ESPNIC (11), and other publications (12, 24, 26) endorse peer review, mentoring, development of standardised guidelines, and formal training or fellowship programmes for accreditation of skills but stop short of recommending formal testing of skills. Structured accredited training programmes that deliver neonatal LUS training in this manner include the Certificate of Clinician Performed Ultrasound (CCPU) course, established by the Australasian Society for Ultrasound in Medicine (ASUM) (31). Focused UltraSound in Intensive Care (FUSIC) and Children Acute Thoracic UltraSound (CACTUS) are initiatives pioneered by the UK Intensive Care Society (ICS) and the Paediatric Intensive Care Society (PICS), respectively, in the UK. They are developing a neonatal framework for practice (12). While assessment may be necessary for identifying whether ultrasound training has improved knowledge and skills, high-quality evidence that it translates into learners being able to perform and implement those skills in practice safely is lacking in neonatology. There is evidence that even in the absence of formal assessment, neonatal lung ultrasound training can be delivered by experts by providing short courses followed up with remote mentoring of learners (with peer review of images) in developing countries with positive results (32).

In the absence of a formal skills assessment, a key question in this study was whether the training improved confidence in diagnosis and made a difference in clinical practice, allowing clinical interventions. The results in Tables 1 and 2 support such a conclusion. Confidence improved both in terms of overall diagnosis and interpretation of the different lung pathologies.

We also looked at the timeline taken for participants to gain confidence. Most participants gained confidence in the diagnoses of different lung pathologies 6 months after the course. Learning lung ultrasound requires time and commitment to understand various disease states. The recent European survey of lung ultrasound practices in European neonatal intensive care units highlights that current lung ultrasound courses may not be sufficient to increase lung ultrasound competencies because a short training period is insufficient to acquire skills to sustain implementation. They also propose dual courses that provide short training followed by a longer practical period with supervised mentorship.

A point of note in this study is that despite clinicians’ confidence in diagnoses of lung pathology using LUS, approximately 70% of them felt they still needed a chest X-ray before implementing treatment decisions. This gap between confidence in diagnosing and implementing treatment warrants special attention. We hypothesise a few reasons for this. It might imply a longer timeline in developing confidence for implementing treatment versus just making a diagnosis. It must also be remembered that not all clinicians in neonatal units will be performing lung ultrasound. Chest X-rays might be needed for interpretation by colleagues who are not performing and cannot interpret lung ultrasounds. They may also be required for serial follow-up and medicolegal reasons.

The fact that a proportion of learners have wanted to do chest X-rays where they found lung ultrasound findings confusing is significant as it demonstrates that they were working within their limitations, opting to default to a chest X-ray (their original standard) to aid interpretation. This also reflects that changing human behaviour and traditional practices, as well as adopting newer technology, requires time and evolution. We must also recognise that lung ultrasound has limitations and cannot be used to diagnose all lung pathologies. In particular, lung ultrasound cannot accurately diagnose disease states such as congenital pulmonary airway malformation, pulmonary bullae, subcutaneous emphysema, and pulmonary and interstitial emphysema. We cannot quantify the volume of the pneumothorax accurately using LUS. A chest X-ray will still be needed to evaluate these conditions, with even CT and/or MRI, if necessary. Antenatal diagnosis, history, clinical presentation, and clinical correlation are vital in deciding the primary radiological modality. Chest X-rays or additional radiological modalities may be needed if ultrasound appearances are confusing or incompatible with the clinical presentation (33). These principles should be part of and incorporated into guidelines for training and credentialing. Although neonatal lung ultrasound gets ingrained in practice, this ensures patient safety.

The current study has some limitations. A significant limitation is the need for a standardised, validated method for formally assessing clinicians’ didactic knowledge and competencies during and after course completion. Hence, the study relies on clinicians’ self-reported confidence levels based on the questionnaire. This has the potential to introduce bias and inaccuracies. Further research is needed to evaluate how best assessment can be integrated to enhance the acquisition of competencies in neonatal lung ultrasound.

We also recognise that the learners were a heterogeneous group of healthcare professionals consisting of trainees and established clinicians working in differing units with different pathologies. This might have affected the participants’ learning curve, potentially impacting results.

Studies of online ultrasound training can improve candidate performance while being time- and cost-effective. This is through reducing the time and money spent organising venues, machines, teachers, phantoms, and/or simulators. They also allow for a low teacher:student ratio, allowing students to train in lung ultrasound globally without the need to travel while they gain practical experience in their institutions through approved pathways. The availability of technology, the Internet, and time commitment are essential in delivering and using such education (14, 30, 34, 35).

In-person lung ultrasound training has the advantages of practical, hands-on, experiential training and learning through simulators and phantoms. The hands-on approach may allow learning of probe use and dexterity and in-person image optimisation through scanning in real time. Such training is unavailable globally, particularly in low- and middle-income countries.

The best way to train learners in lung ultrasound still needs to be discovered. Standardised recommendations for education and certification based on currently available research evidence are complex. A systematic review of the published literature proposes curriculum-based theoretical learning alongside practical hands-on training. They also recommend demonstrating competencies and feedback with a trained instructor who uses some form of assessment to decide when the learner can independently perform scans (30).

A European consortium proposes dual courses with short training followed by more extended follow-up practical training under supervision as well as the production of an international guideline to standardise training. International frameworks for practice, recommendations (10–12, 26), and guidelines (36–38) to standardise practice in neonatal lung ultrasound already exist. Colleagues teaching lung ultrasound must also understand that learners learn differently, as espoused in the Kolb cycle. Providing peer support after course completion (to allow experiential learning) is crucial in developing and taking these skills forward. This could be through face-to-face training, online training, or training through dedicated fellowship programmes depending on their availability, learner needs, location, and availability of expert mentors.

This study demonstrates that the conduct of online modules, with live, online expert guidance, image optimisation critiques, and provision of live video recordings for future reference, enabled physicians from diverse backgrounds to undertake training from experts without the need to travel. Assessments through theoretical and practical testing are desirable to help validate such training (30). To the best of our knowledge, this is the first cross-sectional survey carried out in India to evaluate the utilisation and impact of curriculum-based online training in neonatal LUS. We hope the findings will help develop better training facilities and expertise in this field in Indian NICUs and those in low- and middle-income countries. Further, it demonstrates the learning curve for those wanting to learn neonatal lung ultrasound. There is an urgent need for further studies regarding this, as it may have implications for its uptake and use in neonatal intensive care. By leveraging the programmes of this study, stakeholders can work collaboratively to develop tailored training curricula, establish certification programmes, and improve access to resources and support systems for neonatal POCUS, not just in low- and middle-income countries but globally as well.

## Conclusion

This study establishes that a curriculum-based online training programme with peer review and supervision in neonatal lung ultrasound is feasible. The provision of online training with ongoing peer review and mentorship using a curriculum has the potential to improve the confidence of learners wanting to develop skills in lung ultrasound through distance learning. This model benefits learners who cannot travel and attend face-to-face courses or dedicated lung ultrasound fellowship programmes. It serves as a model for the delivery of neonatal lung ultrasound training in low- and middle-income countries.

## Data Availability

The original contributions presented in the study are included in the article/**Supplementary Material**, further inquiries can be directed to the corresponding author.
