# Supplementary material for: Efficacy of an online lung ultrasound module on skill acquisition by clinician: a new paradigm
Source: Front Pediatr. 2024 Jun 11;12:1406630. doi: 10.3389/fped.2024.1406630 (PMC11197977; doi:10.3389/fped.2024.1406630)
Supplement: Supplementary file 1 [file Datasheet1.pdf]

Supplementary Material

Efficacy of an online Lung Ultrasound module on skill acquisition by clinician- a new paradigm

Authors: Alok Sharma[1], Gunjana Kumar[2], Rema Nagpal[3], Kirti Naranje [4], Arnab Sengupta[5], Vanitha Jagannath[6], Sonali Suryawanshi[7] Pradeep Suryawanshi[8]

*** Correspondence:** Pradeep Suryawanshi, Department of Neonatology, Bharati Vidyapeeth University Medical College, Hospital, and Research Center, Pune, Maharashtra, India,
e-mail: drpradeepsuryawanshi@gmail.com

Supplementary Data

Supplementary Table 1. Subgroup analysis of the clinicians based on their experience with respect to learning acquisition of lung ultrasound skills.

**Experience years**	% Change in acquisition of skills			P value
	≥100%	1-99%	No improvement	0.65
Less than 10 years	59(47.2%)	11(45.8%)	4(33.3%)	
More than 10 years	66(52.8%)	13(54.2%)	8(66.7%)	
Total	125(100%)	24(100%)	12(100%)	

Supplementary file 1: Questionnaire used for the study.

Neonatal POCUS Survey

1. Background and Demographics

This study is being done to evaluate your learning curve after completion of the Neonatal POCUS course in relation to performing lung and cranial ultrasound. This survey will take about 12-15 minutes.

1. **I am a practicing doctor, or medical specialist working with a license to practice in my area of specialization. I consent to participate in this study and, understand that the information obtained will be part of a research study looking at the impact of POCUS training in the field of neonatology**

○ Yes

○ No

2. **Name (in Capital Letters)**

[ ]

3. **Batch**

○ 1

○ 2

○ 3

○ 4

4. **What level of care does your hospital provide in NICU?**

○ Level 1 (Near term babies and Term Babies-Newborn Nursery)

○ Level 2 (Manage neonates with gestation greater than 32 weeks and 1500 gm Special care nursery)

○ Level 3 (NICU with all facilities for neonatal care with gestation age less than 32 weeks, and weight less than 1500gms with the facility of

conventional and/or high-frequency ventilation, Therapeutic Hypothermia, INO along with advanced imaging)

- ○ Level 4 (Level 3 facilities with subspecialties facility including the capability to provide surgical repair of complex congenital or acquired conditions)

5. **What is your professional role?**

- ○ Consultant Neonatologist (More than 10 years experience after postgraduation)
- ○ Consultant pediatrician with a neonatal interest (More than 10 years experience
- ○ after postgraduation)
- ○ Consultant or Senior Specialist Neonatologist or pediatrician (With 5-10 year's experience after postgraduation)
- ○ Registrar, Senior Resident in Pediatrics, Associate Specialist
- ○ Fellow in Neonatology (IAP (Indian Academy of Pediatrics), NNF (National Neonatology Forum), Overseas, DM)
- ○ Resident Doctor in Pediatrics
- ○ Less than 5 years of experience after postgraduation Other (please specify)

[ ]

6. **Does your current place of work have a USG machine**

- ○ No
- ○ Yes but no access to it for the period of the course
- ○ Yes, and I have full access to train with it for the period of the course and thereafter

7. **Did your institution provide an adopted training pathway with accreditation in the neonatal POCUS (cranial, lung, cardiac or vascular access) prior to this POCUS course**

- ○ No formal training pathway

◯ Informal training with no curriculum (See/Learn/Do)

◯ Formal training with a specified curriculum (Accreditation)

8. **Did you have any prior training in POCUS in neonatology before joining this online course of POCUS?**

- ○ Informal theoretical training with no practical training
- ○ Formal training (Online/Face to Face/Other) with practical training without specialist supervision
- ○ Formal practical training (Online/Face to Face/Other) like attending workshops but no specialist supervision later
- ○ Formal practical training (Online/Face-to-face/Other) with specialist supervision and assessment of competencies at the end of the training period
- ○ Informal Practical training

9. **Indicate who primarily performs, interprets and reports Lung US in your institute (before joining this course)**

◯ Paediatric Radiologist

◯ Adult radiologist

◯ Trained neonatologist with accreditation

◯ Trained Neonatologist without certified accreditation

◯ Nurse practitioner

◯ No one

10. Which of the following is the most important driving factor for you to learn and practice lung POCUS? (Rank in order of importance from your perspective)?

≣ ⬍ Self-taught practice after the course is safe and enables care for neonates where facilities for expert radiologists are not available

≣ ⬍ LUS is an upcoming diagnostic modality-Neonatologists doing POCUS themselves could correlate clinically the findings of the baby better and this could help in decision making

≣ ⬍ Evidence-based practice and consensus from experts Globally support the use of POCUS by neonatologists

≣ ⬍ An accredited body in your city/ state/ country or medical council supports the learning of Lung USG

11. Before the course, your experience in performing Lung Ultrasound was

- ◯ Trained neonatologist/ pediatrician - No knowledge and not performing scans
- ◯ Trained neonatologist/pediatrician - Novice level knowledge but not performing scans at all
- ◯ Trained neonatologist/pediatrician - Beginner level of knowledge and performing scans regularly under supervision
- ◯ Trained neonatologist/pediatrician - Beginner level knowledge and performing scans regularly and independently
- ◯ Trained neonatologist/pediatrician - Advanced level knowledge (formal courses) and performing scans regularly independently
- ○ Trained neonatologist/pediatrician -Advanced level knowledge (formal accreditation or training,) and performing scans regularly independently

12.Prior to the course, your ability in diagnosing and reporting Lung US images for Normal Lung and Pneumothorax was at the following level

- ◯ Independently diagnose and report normal lung vs pneumothorax
- ◯ Diagnose and report normal lung, pneumothorax with peer supervision (Radiologist or Trained Neonatologist)
- ◯ Have some knowledge about images but was not confident in
- ◯ diagnosing without chest x-ray Unable to perform LUS

13.How long before you felt confident in diagnosing and reporting normal lung versus pneumothorax after joining the POCUS course?

- ◯ Was already skilled and confident and the course has not so far improved my performance and interpretation of images
- ◯ Was already skilled and confident but the course has improved my performance, interpretation, and reporting of images
- ◯ Felt confident after 3 months of course completion.
- ◯ Felt confident after 6 months of course completion.
- ◯ Felt confident after 12 months of course completion.
- ◯ Still not confident after 12 months of course completion
- ◯ Have not been able to take the training received on the course forwards.

14.Prior to the course, your ability to diagnose and report Lung US images for Normal Lung versus other pathological lung conditions like RDS,TTNB, Pneumonia was at the following level

- ◯ Independently diagnose and report normal lung vs pathological lung conditions
- ◯ Diagnose and report normal and abnormal lung images but with peer supervision (Radiologist or Trained Neonatologist)
- ◯ Have some knowledge about images but was not confident in diagnosing and reporting
- ◯ Unable to perform LUS

15. How long before you felt confident in diagnosing and reporting normal lung versus pathological lung conditions (Including RDS, TTNB,

Pneumonia and others), <u>after joining</u> this online POCUS course on lung ultrasound?

- ◯ Was already skilled and confident and the course has not so far improved my performance, interpretation, and reporting of images
- ◯ Was already skilled and confident but the course has improved my performance, interpretation, and reporting of images
- ◯ Felt confident 3 months after course completion.
- ◯ Felt confident 6 months after course completion.
- ◯ Felt confident 12 months after course completion.
- ◯ Still not confident after 12 months of training
- ◯ Have not been able to take the training received on the course forwards

15. Prior to the course your confidence in diagnosis and assessment of the following LUS pathology was

	Not confident in making the diagnosis,	Some confidence in making the diagnosis but need to confirm the images with an expert always	Confident in making the diagnosis but might need confirmation of images with an expert	Confidence making the diagnosis independently without supervision	Confident in making the diagnosis independently and supervising others
Diagnosis of Pneumothorax	◯	◯	◯	◯	◯
Diagnosis and differentiation of RDS, TTNB	◯	◯	◯	◯	◯

Diagnosis of Consolidation and Pneumonia	○	○	○	○	○
Diagnosis of Collapse /Atelectasis	○	○	○	○	○
Functional LUS-LUS scoring for RDS	○	○	○	○	○
Diaphragmatic Assessment	○	○	○	○	○

16. After completion of this course your confidence in diagnosis and assessment of the following LUS pathology was

	Not confident in making the diagnosis,	Some confidence in making the diagnosis but need to confirm the images with an expert always	Confident in making the diagnosis but might need confirmation of images with an expert	Confidence making the diagnosis independently without supervision	Confident in making the diagnosis independently and supervising others
Diagnosis of Pneumothorax	○	○	○	○	○
Diagnosis and differentiation of RDS, TTNB	○	○	○	○	○
Diagnosis of Consolidation and Pneumonia	○	○	○	○	○
Diagnosis of Collapse /Atelectasis	○	○	○	○	○
Functional LUS-LUS scoring for RDS	○	○	○	○	○
Diaphragmatic Assessment	○	○	○	○	○

18. How confident do you feel with lung POCUS in making treatment decisions in the conditions below:

	Still learning and Not confident in Making a diagnosis And will always need CXR for diagnosis	Confidenet in diagnosis the diseased condition with POCUS but will get a CXR for treatment decisions	Confidenet in diagnosis the diseased start with POCUS but will get a CXR if confused to treat	Confident in making a diagnosis independently and making treatment decisions without CXR
RDS (Giving surfactant)	○	○	○	○
TTNB (Ventilation needs)	○	○	○	○
Pneumonia/Consolidation (Antibiotics)	○	○	○	○
Pneumothorax (Needle thoracentesis or drain)	○	○	○	○
Atelectasis (Lung recruitment maneuvers)	○	○	○	○
Pleural effusion (Needle thoracentesis or drain)	○	○	○	○

Supplementary file 2: Curriculum of Lung Ultrasound module – Total session 8 (each session is of 2 hours, the initial first hour is for reviewing the images by the learners and the next hour is for new topic)

1. Ultrasound machine, Probes, Knobology and Machine Basics, Optimizing the images- Gain, Depth, Focus, zoom, Correct orientation of the probe.

2. Introduction, Indications, and technique of point of care lung USG

3. How to perform LUS- Standard views, Identification of A and B lines, identify lung sliding and seashore sign, Use of M-Mode

4. Common signs on lung USG- Identify B Lines and assess the severity and grading of interstitial lung disease. Comet Tail sign, absent lung sliding, Stratosphere/Bar code sign, seashore signs, lung point versus double lung point, shred sign.

5. Specific condition-
    a. Respiratory distress syndrome- Diagnosis and reporting, Severity scoring, Scoring based surfactant administration.
    b. TTNB- diagnosis and reporting, severity, pleural effusion, double lung point
    c. Pneumonia- Identification, shred sign, air bronchogram
    d. Differentiation between – RDS, Pneumonia and TTNB

6. a. Pneumothorax- Detection on greyscale and M-Mode
   b. Atelectasis
   c. Meconium Aspiration Syndrome- Variable presentation, Lung pulse, atelectasis

7. a. Pleural effusion
   b. Congenital cystic lesion of lung
   c. Diaphragmatic assessment- movement and detection of abnormality
   d. Miscellaneous- pulmonary edema, pulmonary emphysema

e. Research in Lung Ultrasound

8. Image demonstration, clarification of doubts and revision session
